# Higher dietary diversity and appropriate gestational weight gain reduce the risk of low birth weight: a prospective cohort study

**DOI:** 10.1186/s12937-025-01130-8

**Published:** 2025-10-06

**Authors:** Nahla Al-Bayyari, Ana Baylin, Andrew Jones, Marah Hailat

**Affiliations:** 1 Department of Nutrition and Food Processing, Faculty of Al-Huson University College, Al-Balqa Applied University, Al-Salt, Jordan; 2 Department of Nutritional Sciences, School of Public Health, University of Michigan, Ann Arbor, MI USA; 3 Department of General Medicine, Faculty of Medicine, Yarmouk University, Irbid, Jordan

**Keywords:** Dietary diversity, Gestational weight gain, Singleton pregnancy, Birth weight

## Abstract

**Background:**

Low dietary diversity can contribute to undernutrition, impacting gestational weight gain (GWG) and increasing the risk of low birth weight (LBW).

**Objective:**

This study investigates the relationships between maternal dietary diversity, dietary quality, GWG, and LBW in a cohort of singleton pregnant mothers in Jordan. It was hypothesized that higher dietary diversity and appropriate GWG would correlate with a reduced likelihood of LBW and that "minimum dietary diversity for women (MDD-W)" and "prime diet quality scores (PDQS) " would have both indirect and direct effects on birth weight, mediated by GWG.

**Methods:**

The prospective study involved 198 singleton pregnant mothers aged 19 to 45, segmented into three groups by trimester (66 women per trimester). Dietary diversity was assessed using the MDD-W and the PDQS. GWG was classified as appropriate, excess, or inadequate based on pre-pregnancy body mass index (BMI). Birth weights, lengths, and head circumferences of neonates were measured.

**Results:**

Mothers with MDD-W > 5 and PDQS > 21 had significantly higher average birth weights and lengths compared to those with lower scores (MDD-W: 3.1 ± 0.6 vs. 2.6 ± 0.5 kg; PDQS: 3.0 ± 0.6 vs. 2.6 ± 0.5 kg; MDD-W: 49.8 ± 1.7 vs. 48.1 ± 1.7 cm; PDQS: 49.2 ± 1.8 vs. 48.1 ± 1.8 cm). Significant predictors of LBW included GWG for pre-pregnancy BMI, previous LBW deliveries, PDQS, and family income. Inadequate GWG was significantly associated with LBW. GWG significantly mediated the relationship between MDD-W (B = 0.067, *P* < 0.001, 95% CI [0.059–0.076]), PDQS (B = 0.069, *P* < 0.001, 95% CI [0.06–0.077]), and birth weight. Each score increase in MDD-W was associated with a 0.141 kg increase in birth weight (B = 0.141, *P* < 0.001, 95% CI [0.093–0.189]), compared to a 0.041 kg increase for each PDQS score (B = 0.041, *P* < 0.001, 95% CI [0.025–0.058]).

**Conclusions:**

Our findings indicated that both MDD-W and PDQS are associated with birth weight, with higher scores correlating with increased GWG and birth weight. Notably, dietary diversity and GWG relative to pre-pregnancy BMI emerged as robust predictors of birth weight at delivery.

**Supplementary Information:**

The online version contains supplementary material available at 10.1186/s12937-025-01130-8.

## Introduction

Low birth weight (LBW) presents a significant public health challenge, often linked with preterm birth (PTB) and intrauterine growth restriction, which are crucial determinants of neonatal mortality [1–4]. Globally, LBW affects 14.6% of births, while PTB impacts 10%, with higher rates in developing regions [4–6]. In Jordan, rates of LBW (11.8%), PTB (10.5%), and small for gestational age (SGA) (10.0%) have been reported [7].

Despite increased nutritional needs during pregnancy [8], insufficient micronutrient intake is common among women in Africa, Asia, and Latin America [9, 10]. Particularly in developing countries, pregnant women face elevated malnutrition risks due to diverse factors such as inadequate intake of food and socio-economic barriers [11–13]. Maternal malnutrition and inadequate gestational weight gain (GWG) are primary risk factors for LBW. Inappropriate GWG, whether insufficient or excessive, is connected with negative outcomes for both mothers and newborns [14–17].

Low dietary diversity can contribute to undernutrition and impacting GWG. Maternal underweight and low diet diversity have been linked to an increased risk of LBW [18]. During pregnancy, insufficient dietary diversity may heighten the risk of LBW, especially in developing countries [19]. Existing evidence suggests an inverse association between the pregnant mother’s dietary diversity and outcomes like LBW, PTB, and SGA [20, 21], although challenges exist due to sample size variations and measurement methods.

Limited prospective evidence supports the association between maternal dietary diversity, measured using the “ Minimum Dietary Diversity for Women (MDD-W)” indicator, which serves as a proxy for micronutrient adequacy, and reduced risks of adverse pregnancy outcomes [22, 23]. Studies employing “Prime Diet Quality Score (PDQS)” indicator, which scores food grounded into healthy and unhealthy food groups, affirm its influence on pregnancy outcomes, including PTB and LBW, in Sub-Saharan Africa [22]. Moreover, a higher tertile PDQS was linked to a lower risk of LBW and inappropriate GWG, while a middle tertile PDQS was correlated with a lower risk of preterm birth in urban Tanzania [24]. Therefore, considering GWG as a mediator between mothers’ diet and birth outcomes [25], and understanding its impact on pregnancy complications is crucial [26].

In Jordan, research on pregnant mother's dietary intake is scarce, with no exploration of associations between dietary diversity, GWG, and LBW as pregnancy outcomes. Therefore, the primary objective of this study is to investigate the connections between pregnant mothers’ dietary diversity, dietary quality, and LBW within a cohort of singleton pregnant mothers in Jordan and the secondary objective is to determine whether theses connections are mediated by GWG. We hypothesize that higher diet diversity among pregnant mothers with appropriate GWG would correlate with reduced likelihood of LBW and both MDD-W and PDQS have indirect and direct effects on birth weight, mediated by GWG.

## Methods

### Study design and participants recruitment

The study, carried out between August 2022 and June 2023, employed a prospective cohort design focusing on singleton pregnancies. The research took place at antenatal care clinics (ANCs) located within maternal and child health (MCH) centers of the Jordanian Ministry of Health in Northern Jordan. The participants consisted of Jordanian mothers aged between 19 and 45, pregnant with a singleton pregnancy, irrespective of gestational age. Exclusions from the study comprised “pregnant mothers diagnosed with pre-eclampsia, gestational diabetes, autoimmune disorders, chronic diseases (such as diabetes mellitus, liver, and renal diseases), hyperemesis gravidarum, and those with unknown pre-pregnancy weight (weight at conception or two weeks before conception or within the first two weeks of gestation)”.

### Sample size determination

In this study, the data used was previously collected, to identify the prevalence of LBW and determine whether pregnant mothers in the North of Jordan achieved the MDD-W. The sample size determination was followed the “infinite population equation, expressed as *n* = z^2^pq/d^2^, where 'n' represents the sample size, 'z' is the value of the 95% confidence level, 'P' is the estimated average prevalence, 'q' is 1 minus 'p', and 'd' is the accepted error reflecting precision around the population mean” [27]. The prevalence of LBW newborns in Jordan, as recorded by Islam et al. [28], was utilized (13.8%). Consequently, the desired sample was 182.8 women.

To enhance the power of analysis and account for potential exclusions from data analysis, the sample size was increased by at least 10%, resulting in a total of 198 participants.

All ANCs in the northern Jordan were assigned numbers, and six were randomly chosen using a computer random generator of numbers. Study participants were randomly selected by choosing pregnant mothers with odd-numbered orders. Additionally, the selected pregnant mothers were intentionally and randomly stratified into three groups according to their gestational weeks (GWs): first (0–13), second (14–26), and the third (27–40) trimester. To ensure balanced representation across all stages of pregnancy, an equal number of participants (*n* = 66) were selected for each trimester group. This stratification allowed for a more comprehensive analysis of dietary intake, weight gain, and birth outcomes across different stages of pregnancy.

### Sociodemographic and medical data

Data was obtained directly from expectant mothers through individual interviews utilizing a validated questionnaire. Sociodemographic information encompasses “the current age of the mother, the age at marriage, the educational and employment status of both the mother and her spouse, monthly family income, religion, number of family members, health insurance, place of residence, and housing type”. The collected medical data encompass details such as “the last menstrual period, gestational age, miscarriages, stillbirths, low birth weight, parity, spacing between pregnancies, previous deliveries, medications, and supplements such as the iron, calcium and folic acid, and any food allergies or intolerance”. Furthermore, “the history of chronic diseases, gestational hypertension, gestational diabetes, and pre-eclampsia/eclampsia”, were also collected to determine eligibility.

### Determining gestational age

For the majority (96%) of the participating mothers, gestational age (GA) was determined using the date of their last menstrual cycle or period (LMP). In cases where pregnant mothers couldn't recall their LMP date and/or were breastfeeding at conception, the obstetrician assessed GA using ultrasound measurements of the “fetal biparietal diameter (BPD), abdominal circumference(AC), and femur length (FL)”. The GA was determined for eight pregnant mothers (4%) out of the total (*N* = 198) using the aforementioned method.

### Determining and assessing the GWG

The assessment of GWG relied on the anthropometrics of the pregnant mother's weight and height. A stadiometer was employed for height measurement, with women standing barefoot, minimally clothed, straightening their legs, adhering their heels, placing their arms to the side, relaxing their shoulders, and keeping their heads in the “Frankfort horizontal plane” [29]. Weight was assessed using a calibrated and zero-balanced beam scale, with women standing unassisted at the center of the scale while minimally dressed, barefoot, and looking straight ahead [29]. “Quetelet’s formula [weight (kg)/height (m)^2^]” was applied to calculate body mass index (BMI) [29]. Furthermore, the pre-pregnancy weight of the mother was extracted from her ANC records. GWG during the whole pregnancy period was computed as the difference between the weight measured prior to delivery and the weight prior to pregnancy.

To assess GWG outcomes, the 2009 “Institute of Medicine (IOM) guidelines” were used to define the appropriate GWG according to the mother BMI category before pregnancy. The IOM recommends specific ranges of healthy weight gain for pregnant women classified as “underweight (BMI: < 18.5) (12.5–18.0 kg), normal weight (BMI: 18.5–24.99) (11.5–16.0 kg), overweight (BMI: 25–29.99) (7.0–11.5 kg), and obese (BMI > 30) (5.0–9.0 kg)” [26]. Appropriate GWG was classified if the GWG falls between the recommended range for pre-pregnancy BMI, excessive GWG was classified if it exceeded the recommended upper range, while inadequate GWG was determined if it fell below the recommended lower range.

### Birth outcomes data

All pregnancies included in this study resulted in live births without any preterm occurrences. The study examined outcome characteristics such as gestational age at delivery, newborn gender, head circumference, length, and birth weight. We computed “LBW (birth weight < 2.5 kg), SGA (gender-specific birth weight below the 10th percentile), and large for gestational age (LGA) (gender-specific birth weight above the 90th percentile)” for infants of the same GA based on the “INTERGROWTH-21st reference chart” [30]. Additionally, preterm births were identified as deliveries occurring before 37 weeks of gestational age.

Measuring the length of a newborn involved using an infantometer positioned on a raised flat surface. To enhance leg alignment, the newborn was without diapers during the process. Two nurses participated in the procedure; the lead nurse stood on the side, holding the newborn's legs down with one hand while adjusting the footboard with the other. Simultaneously, the assisting nurse positioned herself at the headboard, ensuring the correct placement of the newborn's head against the fixed headboard. Alignment was verified by ensuring “a vertical line drawn from the ear canal to the lower border of the eye socket was perpendicular to the horizontal board”. The actual measurement was taken by gently positioning the “footboard against the infant's feet, ensuring the soles were flat on the board with toes pointing upwards”. The length was measured to the nearest 0.1 cm.

Accurate measurement of the occipital frontal circumference (OFC) or head circumference (HC), is achieved using a “flexible non-stretchable measuring tape”. The OFC is measured around the head, spanning the most “prominent part at the back (occiput) and just above the eyebrows (supraorbital ridges)”. The tape is placed “above the eyebrows, over the ears, and around the widest part of the back of the head” to determine the maximum circumference. It is pulled snugly to compress the hair and the soft tissues underlying it, with the measurement taken to the nearest 0.1 cm.

To measure the newborn's body weight (WT), a specialized infant weighing scale was utilized. The nude newborn without a diaper was positioned at the middle of the scale tray, and the weight was recorded to the nearest 0.01 kg.

### Dietary diversity

A qualified dietitian conducted dietary assessments on participating mothers, utilizing a single “24-h recall and a quantitative food frequency questionnaire (FFQ) for Jordanian pregnant mothers”, validated in 2020 [31]. The MDD-W indicator and the PDQS were used to evaluate dietary diversity. Mothers provided details on foods consumed, preparation methods, quantities, and times and places of intake over the past 24 h. Food models and measuring tools aided in estimating portion sizes. The Food Agriculture Organization in 2016 [32] developed the MDD-W to assess micronutrient sufficiency in reproductive-age women, with a score based on consuming at least five specified food groups out of ten [22]. Each consumed food group received 1 point, with the total determining the mother’s total diet diversity score out of ten. The total dietary diversity score was classified into two categories: one denoting “dietary diversity (consumption of > 5 food groups)” and the other indicating a lack of “dietary diversity (consumption of < 5 food groups)”.

The PDQS comprises fourteen health-promoting food groups, encompassing “dark green vegetables, carrots, cruciferous vegetables, other vegetables, whole citrus fruits, other fruits, whole grains, nuts and seeds, legumes, low‐fat dairy, eggs, fish, poultry, and liquid vegetable oils”. Additionally, seven less favorable (unhealthy) food groups are included, incorporating “red meat, processed meat, potatoes, refined grains, baked goods, fried foods consumed away from home, sugar‐sweetened beverages, and ice cream and desserts”. Fruits and vegetables rich in vitamin A like “pumpkin, passion fruit, apricots, and mango” are integrated into the carrots group [22].

The assignment of foods to the healthy or unhealthy PDQS group is determined by the primary component of mixed dishes. Weekly servings for each food item are calculated by summing daily servings within each food group and then multiplying by seven scoring is based on total weekly servings: “healthy food groups receive 2 points for 4 + servings/week, 1 point for 2–3 servings/week, and 0 points for 0–1 serving/week; unhealthy food groups receive 0 points for 4 + servings/week, 1 point for 2–3 servings/week, and 2 points for 0–1 serving/week”. The cumulative scores for each specified food group contribute to the overall PDQS.

### Statistical analyses

The collected data underwent double entry, verification, and analysis using version 25 of the SPSS statistical package released in 2017. Descriptive statistics, employing frequencies and percentages, were applied to characterize categorical variables, while “means and standard deviations (SD)” were used for continuous variables. The nonparametric “Kolmogorov–Smirnov test” assessed normal distribution for all continuous variables.

Numeric variables such as GWG [26], birth weight for GA, baby WT, length, HC [30], MDD-W [32], and the total PDQS [22] were transformed into categorical variables based on international cutoff values. Associations between “dichotomous and categorical variables were examined using Pearson’s Chi-square (χ^2^) and Fisher’s exact tests”. Pearson correlation explored the relationships between GWG and dietary diversity scores, GA at birth, birth weight for GA, baby gender, WT, length, and HC.

“A Student t-test for independent variables” was conducted to identify significant variations in means among “normally distributed continuous variables”. Sensitivity analyses were performed to investigate whether data collection based on pregnancy trimester interferes with outcomes. Stepwise multiple regression analysis along with logistic regression was conducted to identify the predictors of LBW and the causal relationship. Two mediation analysis models were executed to scrutinize the total, indirect, and direct effects of dietary diversity, the mediator (GWG), and birth weight after adjusting for the potential confounders. The Sobel test calculator determined the *P* value for the indirect effect of both dietary diversity and the mediator on birth weight. *P*- value ≤ 0.05 was used to determine the statistical significance.

## Results

### Participants demographic and anthropometric details

A total of 458 mothers underwent eligibility screening, with 260 failing to meet inclusion criteria and consequently being excluded from the study. The remaining qualified 198 pregnant mothers, based on the inclusion criteria, constituted the cohort analyzed in this study (Supplementary Fig. 1).

“The mean and standard deviation (M ± SD)” for the age, height and pre-pregnancy weight of the study participants were (29 ± 5.9) years, (160.5 ± 5.7) cm, and (65.9 ± 14.0) kg respectively. All mothers identified as Muslims, with 98.5% were married and lived with their husbands. Of the participants, 55.6% lived in rural areas, 25% in rented houses, and the majority (90%) were unemployed. Additionally, 26% reported a family income below 300 JD per month, while 94% had health insurance. About half (48%) had a high level of education, and 48% were assorted as overweight or obese prior to pregnancy.

### Dietary diversity

Assessment of the women’s diet diversity indicated that 96% included starchy grains in their diet the previous day. Additionally, 71%, 73%, and 64% consumed “milk and milk products, meats, and other fruits not rich in vitamin A”, respectively. However, a significant portion, including “66%, 86%, 67%, 80%, 76%, and 53%, did not include legumes, nuts and seeds, eggs, dark green vegetables rich in vitamin A, other fruits, or vegetables rich in vitamin A and other vegetables in their diet. The overall mean (SD) score for the consumed food groups was 4.8 ± 1.6, aligning closely with the threshold for achieving the MDD-W” (Related files Table 1).

**Table 1:** Frequencies of MDD-W, PDQS, PDQSHF and PDQSUF according to the study variables (*N* = 198)

Variable	MDD-W^a^			PDQS^b^				PDQSHF^c^			PDQSUF^d^		
	** < 5**		**≥ 5**	**< 21**	**≥ 21**			**< 14**		**≥ 14**	**< 7**	**≥ 7**	
	**(*n* = 94)**		**(*n* = 104)**	**(*n* = 36)**	**(*n* = 162)**			**(*n* = 116)**		**(*n* = 82)**	**(*n* = 83)**	**(*n* = 115)**	
	**n (%)**		**n (%)**	**n (%)**	**n (%)**			**n (%)**		**n (%)**	**n (%)**	**n (%)**	
Area of residence													
- Urban	50 (53.2)		38 (36.5)	20 (55.6)	68 (42.0)			55 (47.4)		33 (40.2)	42 (50.6)	46 (40.0)	
- Rural	44 (46.8)		66 (63.5)	16 (44.4)	94 (58.0)			61 (52.6)		49 (59.8)	41 (49.4)	69 (60.0)	
	*P* = 0.018			*P* = 0.138				*P* = 0.317			*P* = 0.138		
Housing													
- Owned	68 (72.3)		80 (76.9)	28 (77.8)	120 (74.1)			89 (76.7)		59 (72.0)	57 (68.7)	91 (79.1)	
- Rented	26 (27.7)		24 (23.1)	8 (22.2)	42 (25.9)			27 (23.3)		23 (28.0)	26 (31.3)	24 (20.9)	
	*P* = 0.282			*P* = 0.410				*P* = 0.275			*P* = 0.067		
Mother profession													
- Housewife	89 (94.7)		90 (86.5)	33 (91.7)	146 (90.1)			107 (92.2)		72 (87.8)	71 (85.5)	108 (93.9)	
- Others	5 (5.3)		14 (13.5)	3 (8.3)	16 (9.9)			9 (7.8)		10 (12.2)	12 (14.5)	7 (6.1)	
	*P* = 0.052			*P* = 0.776				*P* = 0.296			*P* = 0.043		
Family monthly income (JD)													
- < 300	26 (27.7)	25 (24.0)		10 (27.8)		41 (25.3)		31 (26.7)		20 (24.4)	23 (27.7)		28 (24.3)
- 301–500	61 (64.9)	71 (68.3)		22 (61.1)		110 (67.9)		76 (65.5)		56 (68.3)	53 (63.9)		79 (68.7)
- > 500	7 (7.4)	8 (7.7)		4 (11.1)		11 (6.8)		9 (7.8)		6 (7.3)	7 (8.4)		8 (7.0
	*P* = 0.843			*P* = 0.606				*P* = 0.918			*P* = 0.771		
Health insurance													
- Yes	87 (92.6)	100 (96.2)		35 (97.2)		152 (93.8)		107 (92.2)		80 (97.6)	82 (98.8)		104 (90.4)
- No	7 (7.4)	4 (3.8)		1 (2.8)		10 (6.2)		9 (7.8)		2 (2.4)	1 (1.2)		11 (9.6)
	*P* = 0.356			*P* = 0.692				*P* = 0.127			*P* = 0.015		
Mother education													
- Secondary and lower	58 (61.7)	46 (44.2)		21 (58.3)			83 (51.2)	65 (56.0)	39 (47.6)		39 (47.0)		65 (56.5)
- Diploma and higher	36 (38.3)	58 (55.8)		15 (41.7)			79 (48.8)	51 (44.0)	43 (52.4)		44 (53.0)		50 (43.5)
	*P* = 0.013			*P* = 0.440				*P* = 0.239			*P* = 0.184		
Iron pills intake													
- Yes	47 (50.0)	44 (42.3)		19 (52.8)			72 (44.4)	54 (46.6)	37 (45.1)		43 (51.8)		48 (41.7)
- No	47 (50.0)	60 (57.7)		17 (47.2)			90 (55.6)	62 (53.4)	45 (54.9)		40 (48.2)		67 (58.3)
	*P* = 0.173			*P* = 0.235				*P* = 0.479			*P* = 0.104		
Main meals intake													
- Yes	39 (41.5)	52 (50.0)		19 (52.8)			72 (44.4)	52 (44.8)	39 (47.6)		46 (55.4)		45 (39.1)
- No	55 (58.5)	52 (50.0)		17 47.2)			90 (55.6)	64 (55.2)	43 (52.4)		37 (44.6)		70 (60.9)
	*P* = 0.154			*P* = 0.235				*P* = 0.407			*P* = 0.017		
Number of meals/day													
- Two or less	61 (64.9)	60 (57.7)		22 (61.1)			99 (61.1)	74 (63.8)	47 (57.3)		41 (49.4)		80 (69.6)
- Three or more	33 (35.1)	44 (42.3)		14 (38.9)			63 (38.9)	42 (36.2)	35 (42.7)		42 (50.6)		35 (30.4)
	*P* = 0.299			*P* = 1.00				*P* = 0.357			*P* = 0.004		
Breakfast intake													
- Usually	47 (50.0)	71 (68.3)		19 (52.8)			99 (61.1)	63 (54.3)	55 (67.1)		57 (68.7)		61 (53.0)
- Sometimes	32 (34.0)	25 (24.0)		12 (33.3)			45 (27.8)	39 (33.6)	18 (22.0)		17 (20.5)		40 (34.8)
- Rarely or never	15 (16.0)	8 (7.7)		5 (13.9)			18 (11.1)	14 (12.1)	9 (10.9)		9 (10.8)		14 (12.2)
	*P* = 0.024			*P* = 0.652				*P* = 0.162			*P* = 0.064		
Newborn gender													
- Male	46 (48.9)	60 (57.7)		21 (58.3)			85 (52.5)	59 (50.9)	47 (57.3)		46 (55.4)		60 (52.2)
- Female	48 (51.1)	44 (42.3)		15 (41.7)			77 (47.5)	57 (49.1)	35 (42.7)		37 (44.6)		55 (47.8)
	*P* = 0.138			*P* = 0.326				*P* = 0.226			*P* = 0.379		
Birth weight for gestational age													
- Appropriate	37 (39.4)	73 (70.2)		13 (36.1)			97 (59.9)	58 (50.0)	52 (63.4)		49 (59.0)		61 (53.0)
- SGA^e^	54 (57.4)	21 (20.2)		23 (63.9)			52 (32.1)	51 (44.0)	24 (29.3)		28 (33.8)		47 (40.9)
- LGA^f^	3 (3.2)	10 (9.6)		^^^0.5 (0.0)			13 (8.0)	7 (6.0)	6 (7.3)		6 (7.2)		7 (6.0)
	*P* < 0.001			*P* = 0.001				*P* = 0.110			*P* = 0.590		

Data are presented as frequencies (n) and percentages (%)

*P ≤ *0.05 is significant for Chi-Square test or Fisher Exact test

^^^Fisher exact test assuming cell with zero is approximately 0.5

^a^*MDD-W*, minimum dietary diversity-women

^b^*PDQS* Prime diet quality score

^c^*PDQSHF* Prime diet quality score for healthy foods

^d^*PDQSUF* Prime diet quality score for unhealthy foods

^e^*SGA* Small for gestational age

^f^*LGA* Large for gestational age

“The PDQS for healthy food groups indicated that approximately 52%, 76%, 75.8%, 57%, 53%, 59%, 41%, 54%, and 97% of pregnant mothers consumed (0-1 serving/week) of dark green vegetables, cruciferous vegetables, carrots, legumes, nuts and seeds, fish, eggs, whole grains, and low-fat milk and milk products, respectively. Conversely, around 77%, 49%, 55%, 81%, and 96% consumed (> 4 serving/week) of other vegetables, whole citrus fruit, other fruits, poultry, and vegetable oils. The overall mean (SD) score for the weekly consumption of healthy food groups (PDQSHF) was 12.8±3.9”.

Regarding the PDQS of unhealthy food groups, “the results revealed that over 50% of pregnant mothers consumed (> 4 servings/week) of potatoes, ice cream, and sweets. However, 40%, 67%, 56%, 38%, and 50% consumed (0–1 serving/week) of red meat, processed meat, refined grains, and packed foods, sugar sweetened beverages, and fried foods from outside the home, respectively. The overall mean (SD) of the weekly consumption of unhealthy food groups score (PDQSUF) was 7.2 ± 2.8”. The total PDQS mean (SD) was 24.7 ± 4.6, and there was a significant correlation (coefficient = 0.577) with the diet diversity score based on the MDD-W indicator (Related files Table 2).

**Table 2:** Comparing means of gestational weight gain and birth outcomes with dietary diversity scores (*N* = 198)

Variable	MDD-W^a^		*P*-value	PDQS^b^		*P*-value
	**< 5**	**≥ 5**		**< 21**	**≥ 21**	
	**(*n* = 94)**	**(*n* = 104)**		**(*n* = 36)**	**(*n* = 162)**	
	**Mean** ± **SD**	**Mean** ± **SD**		**Mean** ± **SD**	**Mean** ± **SD**	
Gestational age	39.1 ± 1.1	39.3 ± 0.8	0.097	39.3 ± 1.1	39.2 ± 1.0	0.643
Gestational weight gain	7.2 ± 10.8	11.1 ± 6.4	0.002	8.5 ± 6.1	10.0 ± 6.0	0.197
Baby body weight	2.6 ± 0.5	3.1 ± 0.6	< 0.001	2.6 ± 0.5	3.0 ± 0.6	0.003
Baby length	48.1 ± 1.7	49.8 ± 1.7	< 0.001	48.1 ± 1.8	49.2 ± 1.8	0.001
Baby head circumference	34.4 ± 0.9	35.0 ± 1.0	< 0.001	34.6 ± 1.1	34.7 ± 1.0	0.366

Data are presented as means ± standard deviations (SD)

*P*-value < 0.05 is statistically significant for student independent samples t-test

^a^*MDD-W* Minimum dietary diversity-women

^b^*PDQS* Prime diet quality score

### Comparing dietary diversity scores with the study variables

Table 1 clarifies significant variations (*P ≤ *0.05) in MDD-W related to the area of residence, mother's education, breakfast intake, and birth weight for gestational age. Rural mothers had higher percentages of MDD-W ≥ 5 in contrast to urban mothers, while less educated mothers had higher percentages of MDD-W < 5 compared to their highly educated counterparts. Consistent breakfast consumption was correlated with higher instances of MDD-W ≥ 5. Conversely, mothers with MDD-W < 5 had higher percentages of infants classified as SAG.

The PDQSUF also varied statistically and significantly with mother's profession, main meal consumption, and number of meals per day. Specifically, housewives exhibited increased proportions of PDQSUF ≥ 7, indicative of lower consumption of unhealthy food groups. Moreover, mothers who skipped daily main meals or consumed fewer than two meals per day showed higher frequencies of PDQSUF groups ≥ 7.

### Dietary diversity, GWG and birth outcomes

Sensitivity analyses showed no significant differences in the dietary diversity based on pregnancy trimester. Table 2 illustrates the results of independent samples t-tests conducted on GA, GWG, and birth outcomes of length, weight, and head circumference, categorized by dietary diversity based on both the MDD-W and total PDQS. The (means ± SD) of gestational age was above 39 weeks, with no preterm births among the study participants. Additionally, no significant differences were found based on dietary diversity. The mean of GWG (11.1 ± 6.4 vs. 7.2 ± 10.8), birth weight (3.1 ± 0.6 vs. 2.6 ± 0.5), length (49.8 ± 1.7 vs. 48.1 ± 1.7), and head circumference (35.0 ± 1.0 vs. 34.4 ± 0.9) were significantly higher for mothers with MDD-W ≥ 5 compared to those with MDD-W < 5. Additionally, the (means ± SD) of birth outcomes, including birth weight (3.0 ± 0.6 vs. 2.6 ± 0.5) and length (49.2 ± 1.8 vs. 48.1 ± 1.8), exhibited statistically significant differences between mothers scoring total PDQS ≥ 21 and those scoring total PDQS < 21.

Table 3 presents the outcomes of a comparison involving dietary diversity, iron supplement intake, and birth outcomes categorized by GWG for pre-pregnancy BMI. Significantly distinct results (*P ≤ *0.05) were observed in relation to iron pill consumption, MDD-W, and various outcomes of birth, including newborn birth WT, length, HC, and birth weight for GA. Mothers who did not take iron supplements and had an MDD-W < 5 exhibited higher proportions of inadequate GWG. Furthermore, among mothers with inadequate GWG for pre-pregnancy BMI, 60%, 92%, 76%, and 77% delivered babies with birth “weight at birth < 2.5 kg, length < 50 cm, head circumference < 35 cm”, and SGA, respectively.

**Table 3:** Maternal dietary diversity scores, and birth outcome according to gestational weight gain for pre-pregnancy BMI (*N* = 198)

Variable	Gestational weight gain for pre-pregnancy BMI			*P*-Value
	**Appropriate**	**Excessive**	**Inadequate**	
	**(*n* = 61)**	**(*n* = 58)**	**(*n* = 79)**	
	**n (%)**	**n (%)**	**n (%)**	
Iron pills intake				
- Yes	34 (55.7)	31 (53.4)	26 (32.9)	0.011
- No	27 (44.3)	27 (46.6)	53 (67.1)	
MDD-W^a^				
- < 5	21 (34.4)	21 (36.2)	52 (65.8)	< 0.001
- ≥ 5	40 (65.6)	37 (63.8)	27 (34.2)	
PDQSHF^b^				
- < 14	31 (50.8)	32 (55.2)	53 (67.1)	0.126
- ≥ 14	30 (49.2)	26 (44.8)	26 (32.9)	
PDQSUF^c^				
- < 7	27 (44.3)	26 (44.8)	30 (38.0)	0.656
- ≥ 7	34 (55.7)	32 (55.2)	49 (62.0)	
Total PDQS^d^				
- < 21	9 (14.8)	8 (13.8)	19 (24.1)	0.216
- ≥ 21	52 (85.2)	50 (86.2)	60 (75.9)	
Baby gender				
- Male	30 (49.2)	37 (63.8)	39 (49.4)	0.176
- Female	31 (50.8)	21 (36.2)	40 (50.6)	
Baby body weight at birth				
- < 2.5 kg	3 (4.9)	1 (1.7)	47 (59.5)	< 0.001
- ≥ 2.5 kg	58 (95.1)	57 (98.3)	32 (40.5)	
Baby length at birth				
- < 50 cm	26 (42.6)	15 (25.9)	73 (92.4)	< 0.001
- ≥ 50 cm	35 (57.4)	43 (74.1)	6 (7.6)	
Baby head circumference at birth				
- < 35 cm	18 (29.5)	6 (10.3)	60 (75.9)	< 0.001
- ≥ 35 cm	43 (70.5)	52 (89.7)	19 (24.1)	
Birth weight for gestational age				
- Appropriate	51 (83.6)	41 (70.7)	18 (22.8)	< 0.001
- SGA^e^	9 (14.8)	5 (8.6)	61 (77.2)	
- LGA^f^	1 (1.6)	12 (20.7)	0.5^^^ (0.0)	

Data are presented as frequencies (n) and percentages (%)

*P ≤ *0.05 is significant for Chi-Square test and/or Fisher exact test

^^^Fisher exact test assuming cell with zero is approximately 0.5

^a^MDD-W, minimum dietary diversity-women

^b^PDQSHF, prime diet quality score for healthy foods

^c^PDQSUF, prime diet quality score for unhealthy foods

^d^PDQS, prime diet quality score

^e^SGA, small for gestational age

^f^LGA, large for gestational age

The Pearson correlation coefficients revealed a significant positive correlation (*P* = 0.01) between GWG and various factors, including MDD-W, PDQSHF, total PDQS, GA at birth, as well as outcomes of birth such as birth WT, length, HC, and birth weight for GA (Table 4).

**Table 4:** Pearson correlation coefficients between gestational weight gain, dietary diversity scores, gestational age at birth, and birth outcomes (*N* = 198)

Variable	Correlation coefficient
Gestational weight gain	1
MDD-W^a^	0.215^**^
PDQSHF^b^	0.201^**^
PDQSUF^c^	-0.117-
Total PDQS^d^	0.171^*^
Gestational age at birth	0.247^**^
Baby gender	-0.115-
Baby body weight at birth	0.751^**^
Baby length at birth	0.645^**^
Baby head circumference at birth	0.602^**^
Birth weight for gestational age	0.399

*Pearson correlation is significant at the 0.05 level (2-tailed), ^**^Pearson correlation is significant at the 0.01 level (2-tailed),

^a^*MDD-W* Minimum dietary diversity-women

^b^*PDQSHF* Prime diet quality score for healthy foods

^c^*PDQSUF* Prime diet quality score for unhealthy foods

^d^*PDQS* Prime diet quality score

A stepwise multiple regression was conducted to identify predictors of LBW. Independent variables of GWG for pre-pregnancy BMI, MDD-W, total PDQS, previous LBW deliveries, iron pill intake, family monthly income, area of residence, mother's profession, mother's education, hemoglobin, and ferritin levels were included in the model. The model yielded a significant result, F(1, 193) = 4.142, *P* = 0.043, *R*^2^ = 0.358. Further examination of individual predictors revealed that GWG for pre-pregnancy BMI (B = 0.282, *P* < 0.0001, 95% CI [0.222–0.342]), previous LBW deliveries (B = 0.247, *P* = 0.003, 95% CI [0.085–0.410]), total PDQS (B = -0.159, *P* = 0.017, 95% CI [-0.289- -0.029]), and family monthly income (B = -0.094, *P* = 0.043, 95% CI [-0.185—-0.003]) were significant predictors of LBW (Supplementary Table 1).

As presented in Supplementary Table 2, family monthly income, history of previous LBW pregnancies, GWG for pre-pregnancy BMI, and dietary diversity measured by total PDQS (but not MDDS) significantly (*P* < 0.05) contributed to the binary logistic regression model for the birth weight outcome. Decreases in total PDQS and family monthly income were correlated with higher odds of low birth weight, with a similar trend observed for MDDS, although not reaching statistical significance. Conversely, elevated odds of LBW were correlated with inadequate GWG and a history of multiple previous LBW pregnancies. The odds ratios indicated that women with a higher total prime diet quality score (TPDQS ≥ 21) were significantly less likely to have a LBW baby (*P* = 0.039), with a reduction in risk of approximately 70%. Similarly, women with a higher family monthly income were significantly less likely to have a LBW baby (*P* = 0.037), with a risk reduction of about 58%. Additionally, each additional previous LBW pregnancy and insufficient GWG elevated the likelihood of delivering a LBW baby by 10.2 and 12.1 times, respectively.

### Indirect and direct effects of dietary diversity and GWG on birth weight outcome

Simple mediation analysis for Andrew F. and Hayes PROCESS procedure for SPSS version 4.2 model 4 was utilized. The coefficient of the dietary diversity as either MDD-w or PDQS (predictor or the independent variable) on the mediator (GWG) is the “a” path. The coefficient of the mediator (GWG) on the birth weight (outcome or the dependent variable) is the “b” path. The indirect effect is the coefficient of the “a” path multiplied by the coefficient of the “b” path. The sum of indirect and the direct effect is considered as the total effect.

Mediation analysis revealed notable findings regarding the impact of the diversity of the diet, as measured by both the MDD-W-model 1 and PDQS-model 2, on the mediator (GWG) and subsequently on birth weight.

The effect of dietary diversity on the mediator was statistically significant (B = 0.827, *P* = 0.002, 95% CI [0.299–1.355]) for MDD-W-mode l and for PDQS-model 2 (B = 0.233, *P* = 0.016, 95% CI [0.041–0.404]). Similar statistically significant differences were noticed in the effect of the mediator (GWG) on birth weight in both models, the MDD-W-mode l (B = 0.067, *P* < 0.001, 95% CI [0.059–0.076]) and the PDQS-model 2 (B = 0.069, *P* < 0.001, 95% CI [0.06–0.077]).

Considering the total effect, in model 1, each score increase in the MDD-W was associated with 0.141 kg increase in birth weight (B = 0.141, *P* < 0.001, 95% CI [0.093–0.189]) compared to 0.041 kg increase for each PDQS in model 2 (B = 0.041, *P* < 0.001, 95% CI [0.025–0.058]). The direct effects in both models on birth weight also differed significantly, with the direct effect of MDD-W (B = 0.085, *P* < 0.001, 95% CI [0.052–0.118]) in model 1 compared to PDQS in model 2 (B = 0.026, *P* < 0.001, 95% CI [0.015–0.037]).

In both models the total (ꞵ = 0.382 vs 0.328) and the direct (ꞵ = 0.231 vs 0.206) effects on birth weight was similar when we look at the standardized coefficients respectively.

It is noteworthy that both total (c) and direct (c′) effects were found to be significant. This indicates that GWG partially mediates the relationship between dietary diversity and the birth WT.

Examining the indirect effects, each score increases in MDD-W and PDQS was associated with an average birth weight increase of 0.056 and 0.015 kg, respectively. These effects were statistically significant for both MDD-W (B = 0.056, *P* = 0.002, 95% CI [0.018–0.094]) and PDQS (B = 0.015, *P* = 0.017, 95% CI [0.004–0.026]). Notably, the indirect effect of MDD-W on birth weight was similar to that of the PDQS when we look at the standardized coefficients (ꞵ = 0.151 and 0.122) respectively (Table 5).

**Table 5:** Direct and indirect effect of dietary diversity in terms of MDD-W and PDQS on the birth weight (*N* = 198)

	**Unstandardized coefficients**		**Standardized coefficients**			**95% CI for B**	
**Effect**	**B**	**SE(B)**	**ꞵ**	**t**	***p***	**Lower**	**Upper**
***Model 1***							
a: MDD-W—> gestational weight gain	0.827	0.268	0.215	3.089	0.002	0.299	1.355
b: gestational weight gain—> birth weight	0.067	0.004	0.701	15.38	< 0.001	0.059	0.076
c (total): MDD-W—> birth weight	0.141	0.024	0.382	5.781	< 0.001	0.093	0.189
c’ (direct): MDD-W—> birth weight	0.085	0.017	0.231	5.063	< 0.001	0.052	0.118
ab (indirect): MDD-W—> gestational weight gain—> birth weight	0.056	0.019	0.151	3.030	^*^0.002	0.018	0.094
***Model 2***							
a: PDQS—> gestational weight gain	0.223	0.092	0.171	2.423	0.016	0.041	0.404
b: gestational weight gain—> birth weight	0.069	0.004	0.715	15.65	< 0.001	0.060	0.077
c (total): PDQS—> birth weight	0.041	0.009	0.328	4.866	< 0.001	0.025	0.058
c’ (direct): PDQS—> birth weight	0.026	0.006	0.206	4.515	< 0.001	0.015	0.037
ab (indirect): PDQS—> gestational weight gain—> birth weight	0.015	0.006	0.122	2.400	^*^0.017	0.004	0.026

Both models were adjusted for previous low birth weight pregnancies, iron bills intake, mothers’ educational level, mother’s professional, family monthly income, area of residence, and health insurance

*P ≤ *0.05 is statistically significant

*CI* Confidence interval, *MDD-W* Minimum dietary diversity-women, *PDQS* Prime diet quality score

^*^P value for indirect effect is calculated by Sobel test

## Discussion

This study prospectively examined the effect of dietary diversity and GWG on the outcomes of birth in a cohort of healthy singleton pregnancies in Northern Jordan. More than half of mothers (52.5%) achieved a minimally diverse diet and 82% achieved a total diet quality score above 21. The MDD-W is affected by the residential area and the mothers’ educational level. In our study, rural residential and higher education levels showed higher MDD-W. Recently, a systematic review and meta-analysis involving 9230 pregnant women from Ethiopia reported a pooled prevalence of inadequate dietary diversity at 53% [33], similar to our findings. Furthermore, the review highlighted rural residence and lack of formal education as contributing factors to inadequate dietary diversity [33]. While our results corroborate the impact of education, they differ concerning residency. Contrary to expectations, our study revealed that pregnant mothers residing in rural areas exhibited higher dietary diversity. This phenomenon can be attributed to the lower food prices in rural regions as opposed to urban areas. For instance, the prices of milk and milk products in urban areas are nearly double those in rural areas. Yang et al. [23] reported that pregnant mothers with higher MDD-W scores were more likely to have proficient occupations and tended to have higher PDQS scores. Our findings align with theirs, although we observed that housewives or unemployed mothers tend to have higher mean scores on PDQSUF. Similarly, prior research indicates that consuming breakfast is linked to increased dietary diversity scores, as shown by Aminianfar et al. [34]. However, our findings diverge from this trend, as women who do not consume their main three meals exhibited higher PDQSUF scores.

Contrary to previously published studies [35, 36]. This discrepancy could be explained by pregnant women avoiding unhealthy food groups and focusing on healthier options, especially when they are not consuming their usual three main meals.

The current study showed a significant connection between higher MDD-W and PDQS with a reduced incidence of SGA babies. Moreover, these women exhibited higher rates of GWG and favorable birth outcomes in terms of length, weight, and head circumference. A recent investigation explored the correlations between dietary diversity of mothers, GWG and outcomes of birth among participants in a Tanzanian trial. Notably, MDD‐W did not exhibit any significant associations with either GWG or the outcomes of birth. Conversely, for PDQS, a comparison between the lowest and highest tertiles indicated a decreased risk of inappropriate GWG and preterm birth among mothers in the latter group. Furthermore, upon excluding mothers with previous complications, a higher total PDQS was correlated with a reduced risk of LBW as an adverse birth outcome [23]. Given that SGA babies, known as “birth weight below the 10th percentile”, fall within the LBW category, this finding substantiates the notion. However, while we concur with the observed relationship between PDQS and birth outcomes, we challenge the absence of correlation between MDD-W, GWG, and outcomes of birth. Another study discovered that higher scores indicating diverse dietary intake were connected with a decreased prevalence of SGA infants, while a diverse PDQS was linked to a reduced likelihood of PTB, LBW, and pregnancy loss. These findings indicate that both the diversity and quality of diet are crucial in understanding the dietary risk factors linked to adverse birth outcomes [22].

Karimi et al. [18] discovered an association between maternal diverse dietary intake and nutritional sufficiency prior to and during pregnancy, as well as anthropometric measurements of neonates upon birth. Our results corroborate these findings, particularly evident when analyzing the anthropometric measurements of length, weight, and head circumference in babies. A notable increase in these anthropometric measurements was observed among women with higher MDD-W and PDQS.

Family monthly income and maternal educational level are recognized as determinants of dietary diversity practice, with higher income and educational levels correlating with adequate dietary diversity [37]. Consequently, family monthly income emerges as an indicator of birth weight outcomes, as evidenced in our study where each unit increase in family income corresponded to an 11% decrease in the risk of LBW. However, previous occurrences of LBW delivers and inadequate GWG relative to pre-pregnancy BMI also prove instrumental in predicting the likelihood of LBW, as reported in this study. Specifically, the odds of LBW decrease by 20% among mothers with higher dietary diversity [38]. Moreover, upon adjusting for pregnant women's baseline characteristics, the study revealed that those with inadequate dietary diversity scores faced an increased risk of LBW [39].

Evidence suggests that variables like the iron intake [40, 41], maternal weight gain during pregnancy [42], preterm birth [43], and maternal anemia [44] are associated with LBW. Insufficient intake of iron hampers the effective transfer of iron to the fetus, disrupts neuronal and hormonal regulation during pregnancy, and impedes fetal oxygenation, thereby affecting growth and development adversely [45]. Our study underscores a significant connection between intake of iron and GWG, mediating the impact of dietary diversity on infant birth weight. Mothers who did not consume iron pills have a higher propensity to experience inadequate GWG and subsequently give birth to LBW infants. This finding aligns with previous research [46, 47], indicating a positive linkage between iron pill compliance and baby’s birth weight.

Severely inappropriate GWG was linked to a heightened hazard of LBW and SGA compared to appropriate GWG. Conversely, the higher likelihood of preterm birth, LGA, and macrosomia compared to appropriate GWG was linked to too-much GWG. The age of the mother and her BMI prior to pregnancy influenced the strength and direction of these associations between GWG adequacy and various neonatal outcomes [48]. In alignment with these findings, our results confirm that the risk of LBW escalates with inadequate GWG relative to pre-pregnancy BMI and an increased number of prior LBW pregnancies.

Diverse intake of diet during pregnancy plays a crucial influence on preventing neonatal LBW by influencing maternal GWG. Research has highlighted that maintaining high dietary diversity throughout pregnancy impacts GWG in a positive manner [49]. Moreover, studies indicate that mothers who experience greater gestational weight gain often deliver infants with greater birth weights [50, 51]. Additionally, through pregnancy adequate diversity of the diet reduces the likelihood of nutrient deficiencies, particularly anemia, in mothers, thereby enhancing fetal growth. Systematic reviews and meta-analyses have consistently shown a significant correlation between anemia among pregnant mothers and LBW in babies [52, 53]. Zerfu et al. [21] proposed that dietary diversity throughout pregnancy correlates with a reduction in the hazards of maternal anemia. In a study cohort comprising 1675 pregnant women, Ghosh et al. [54] found a direct link between dietary diversity and serum hemoglobin levels. However, conflicting results have been reported in previous studies [55]. In our current investigation, we noted that mothers who supplemented with iron demonstrated higher MDD-W and PDQS compared to those who did not.

In their study, Minami et al. [56] inspected the impact of energy intake on birth weight in Japanese singleton pregnancies and discovered that GWG plays a mediating role in this relationship. The findings revealed that transitioning from low to moderate energy intake resulted in an average increase of 13.43 g in birth weight. In this study, GWG partially mediates the impact of dietary diversity on birth weight. Upon examining the indirect effects, each score increases in MDD-W and PDQS were associated with an average birth weight increase of 0.056 and 0.015 kg, respectively. These effects were statistically significant for both MDD-W and PDQS. Our results underscore a logical and discernible pattern; as dietary diversity increases, so does GWG, which in turn significantly mediates the effect on birth weight in a positive manner.

This study boasts several strengths, including its prospective design, which allows for the exploration of the impact of both the MDD-W and the PDQS on the outcomes of birth in a diverse population of singleton pregnancies residing in rural and urban regions. Furthermore, the utilization of mediation analysis facilitated the examination of both the indirect and direct effects of dietary diversity and GWG on birth weight.

The findings of this study supported our hypothesis, although several limitations were noted. The sample size was relatively small, and dietary data, collected via a single 24-h recall at the onset of the mother's enrollment, might not fully capture dietary patterns throughout pregnancy. Additionally, maternal weight gain was not monitored on a monthly basis, potentially impacting the accuracy of GWG assessments. Dietary diversity scores were determined based on the 24-h recall method, which has inherent limitations such as reliance on memory, leading to potential underreporting or overreporting, and may not fully reflect an individual's habitual dietary intake. Moreover, GA estimation relied on the last date of menstrual period, which could introduce inaccuracies due to inaccurate reporting, potentially resulting in nondifferential misclassification of outcomes associated to GA.

It is important to note that the applicability of our results may be restricted to specific populations in the Levant region with similar demographic characteristics.

## Conclusions

In conclusion, our findings indicated that both MDD-W and PDQS are associated with birth weight, with higher scores correlating with increased GWG and birth weight. Notably, dietary diversity and GWG relative to pre-pregnancy BMI emerged as robust predictors of birth weight at delivery.

This study offers valuable insights into the pregnant women dietary intake in Northern Jordan, underscoring the importance of dietary diversity and quality in achieving ideal GWG and favorable outcomes of birth. Further community-based research is crucial for developing effective and influential strategies to assess and promote healthy diets for mothers in practical settings.

## Supplementary Information

Supplementary Material 1: Figure 1. Illustrates the selection process of study participants who met the inclusion criteria and were subsequently included in the analysis.

Supplementary Material 2.

Supplementary Material 3.

## Data Availability

No datasets were generated or analysed during the current study.

## Abbreviations

GWG: Gestational weight gain
LBW: Low birth weight
MDD-W: Minimum dietary diversity for women
PDQS: Prime diet quality scores
BMI: Body mass index
PTB: Preterm birth
SGA: Small for gestational age
ANCs: Antenatal care clinics
MCH: Maternal and child health
GWs: Gestational weeks
GA: Gestational age
LMP: Last menstrual cycle or period
BPD: Fetal biparietal diameter
AC: Abdominal circumference
FL: Femur length
IOM: Institute of Medicine
LGA: Large for gestational age
OFC: Occipital frontal circumference
HC: Head circumference
WT: Body weight
FFQ: Food frequency questionnaire
PDQSHF: Prime diet quality score of healthy food
PDQSUF: Prime diet quality score of unhealthy food
