# Supplementary material for: Higher dietary diversity and appropriate gestational weight gain reduce the risk of low birth weight: a prospective cohort study
Source: Nutr J. 2025 Oct 6;24:151. doi: 10.1186/s12937-025-01130-8 (PMC12502405; doi:10.1186/s12937-025-01130-8)
Supplement: Supplementary file 1 — Supplementary Material 1: Figure 1. Illustrates the selection process of study participants who met the inclusion criteria and were subsequently included in the analysis. [file 12937_2025_1130_MOESM1_ESM.docx]

Assessed for eligibility

(n = 458)

First trimester

(n = 66)

Third trimester

(n = 66)

Second trimester

(n = 66)

Hyperemesis gravidarum

(n = 71)

Hypothyroidism

(n = 32)

Gestational diabetes

(n = 48)

Meet the inclusion criteria

(n = 198)

Did not meet the inclusion criteria

(n = 260)

Missing pre-pregnancy weight

(n = 88)

Autoimmune disorders

(n = 21)
