# Supplementary material for: Higher dietary diversity and appropriate gestational weight gain reduce the risk of low birth weight: a prospective cohort study
Source: Nutr J. 2025 Oct 6;24:151. doi: 10.1186/s12937-025-01130-8 (PMC12502405; doi:10.1186/s12937-025-01130-8)
Supplement: Supplementary file 2 — Supplementary Material 2. [file 12937_2025_1130_MOESM2_ESM.docx]

**Table 1.** Predictors of low birth weight using multiple stepwise regression analysis (N = 198).

|  | **Unstandardized coefficients** | | **Standardized**  **coefficients** |  |  | **95% CI for B** | |
| --- | --- | --- | --- | --- | --- | --- | --- |
| **Model** | **B** | **SE(B)** | **ꞵ** | **t** | **p** | **Lower** | **Upper** |
| ***Model 1*** |  |  |  |  |  |  |  |
| Constant | -0.053 | 0.043 |  | -1.225 | 0.222 | -0.138 | 0.032 |
| Gestational weight gain for pre-pregnancy BMI | 0.284 | 0.031 | 0.544 | 9.073 | <0.001 | 0.223 | 0.346 |
| ***Model 2*** |  |  |  |  |  |  |  |
| Constant | -0.280 | 0.088 |  | -3.166 | 0.002 | -0.454 | -0.106 |
| Gestational weight gain for pre-pregnancy BMI | 0.292 | 0.031 | 0.558 | 9.451 | <0.001 | 0.231 | 0.353 |
| Previous LBW deliveries | 0.245 | 0.084 | 0.173 | 2.926 | 0.004 | 0.080 | 0.410 |
| ***Model 3*** |  |  |  |  |  |  | |
| Constant | -0.135 | 0.107 |  | -1.267 | 0.207 | -0.345 | 0.075 |
| Gestational weight gain for pre-pregnancy BMI | 0.284 | 0.031 | 0.543 | 9.248 | <0.001 | 0.223 | 0.344 |
| Previous LBW deliveries | 0.237 | 0.083 | 0.167 | 2.854 | 0.005 | 0.073 | 0.400 |
| Total PDQS | -0.157 | 0.066 | -0.138 | -2.366 | 0.019 | -0.288 | -0.026 |
| ***Model 4*** |  |  |  |  |  |  |  |
| Constant | -0.064 | 0.111 |  | -0.579 | 0.564 | -0.284 | 0.155 |
| Gestational weight gain for pre-pregnancy BMI | 0.282 | 0.030 | 0.540 | 9.266 | <0.001 | 0.222 | 0.342 |
| Previous LBW deliveries | 0.247 | 0.082 | 0.174 | 2.998 | 0.003 | 0.085 | 0.410 |
| Total PDQS | -0.159 | 0.066 | -0.140 | -2.413 | 0.017 | -0.289 | -0.029 |
| Family monthly income | -0.094 | 0.046 | -0.118 | -2.035 | 0.043 | -0.185 | -0.003 |

P ≤ 0.05 is statistically significant.

Variables excluded from model 1: Area of residence, family monthly income, mother profession, mother education, iron pills intake, previous LBW deliveries, MDD-W, total PDQS, hemoglobin, and ferritin levels.

Variables excluded from model 2: Area of residence, family monthly income, mother profession, mother education, iron pills intake, MDD-W, total PDQS, hemoglobin, and ferritin levels.

Variables excluded from model 3: Area of residence, family monthly income, mother profession, mother education, iron pills intake, MDD-W, hemoglobin, and ferritin levels.

Variables excluded from model 4: Area of residence, mother profession, mother education, iron pills intake, MDD-W, hemoglobin, and ferritin levels.

Abbreviations: CI, confidence interval; BMI, body mass index; LBW, low birth weight; MDD-W, minimum dietary diversity-women; PDQS, prime diet quality score.
