# Supplementary material for: Higher dietary diversity and appropriate gestational weight gain reduce the risk of low birth weight: a prospective cohort study
Source: Nutr J. 2025 Oct 6;24:151. doi: 10.1186/s12937-025-01130-8 (PMC12502405; doi:10.1186/s12937-025-01130-8)
Supplement: Supplementary file 3 — Supplementary Material 3. [file 12937_2025_1130_MOESM3_ESM.docx]

**Table 2.** Binary logistic regression model^1^ for the birth weight outcome.

| **Variable** | **b** | **S.E.** | **P-value** | **OR** | **95% C.I. for OR** | |
| --- | --- | --- | --- | --- | --- | --- |
|  |  |  |  |  | **Lower** | **Upper** |
| Area of residence | 0.393 | 0.445 | 0.377 | 1.48 | 0.619 | 3.542 |
| Family monthly income | -0.860- | 0.413 | 0.037 | 0.423 | 0.188 | 0.950 |
| Previous LBW pregnancies | 2.33 | 0.910 | 0.011 | 10.234 | 1.719 | 60.924 |
| Iron pills intake | 0.374 | 0.456 | 0.413 | 1.453 | 0.594 | 3.553 |
| GWG for pre-pregnancy BMI | 2.50 | 0.444 | 0.000 | 12.057 | 5.047 | 28.804 |
| MDDS-W | -0.079- | 0.483 | 0.869 | 0.924 | 0.359 | 2.379 |
| Total PDQS | -1.194- | 0.577 | 0.039 | 0.303 | 0.098 | 0.939 |
| Constant | -5.625- | 1.289 | 0.000 | 0.004 |  |  |

^1^Hosmer and Lemeshow test: χ² = 9.098; df = 7; *p*-value = 0.246.

B: Regression coefficient. S.E.: Standard error of the regression coefficient.

CI: Confidence Interval. LBW: Low Birth Weight.

GWG: Gestational Weight Gain. MDDS-W: minimum dietary diversity-women.

PDQS: prime diet quality score.

P-value ≤ 0.05 is statistically significant.
